# Differentiation of intestinal tuberculosis and Crohn’s disease through an explainable machine learning method

**DOI:** 10.1038/s41598-022-05571-7

**Published:** 2022-02-02

**Authors:** Futian Weng, Yu Meng, Fanggen Lu, Yuying Wang, Weiwei Wang, Long Xu, Dongsheng Cheng, Jianping Zhu

**Affiliations:** 1 School of Medicine, Xiamen University, Xiamen, 361005 Fujian China; 2 National Institute for Data Science in Health and Medicine, Xiamen University, Xiamen, 361005 Fujian China; 3 Data Mining Research Center, Xiamen University, Xiamen, 361005 Fujian China; 4 Department of Gastroenterology and Hepatology, Shenzhen University General Hospital, Shenzhen, 518055 China; 5 Shenzhen University Clinical Medical Academy, Shenzhen University, Shenzhen, 518037 China; 6 The Gastroenterology Department of Second Xiangya Hospital, Central South University, Changsha, 410011 China; 7 School of Management, Xiamen University, Xiamen, 361005 Futian China; 8 School of Software Engineering, Shenzhen Institute of Information Technology, Shenzhen, 518172 China

**Keywords:** Crohn's disease, Tuberculosis, Computer science

## Abstract

Differentiation between Crohn’s disease and intestinal tuberculosis is difficult but crucial for medical decisions. This study aims to develop an effective framework to distinguish these two diseases through an explainable machine learning (ML) model. After feature selection, a total of nine variables are extracted, including intestinal surgery, abdominal, bloody stool, PPD, knot, ESAT-6, CFP-10, intestinal dilatation and comb sign. Besides, we compared the predictive performance of the ML methods with traditional statistical methods. This work also provides insights into the ML model’s outcome through the SHAP method for the first time. A cohort consisting of 200 patients’ data (CD = 160, ITB = 40) is used in training and validating models. Results illustrate that the XGBoost algorithm outperforms other classifiers in terms of area under the receiver operating characteristic curve (AUC), sensitivity, specificity, precision and Matthews correlation coefficient (MCC), yielding values of 0.891, 0.813, 0.969, 0.867 and 0.801 respectively. More importantly, the prediction outcomes of XGBoost can be effectively explained through the SHAP method. The proposed framework proves that the effectiveness of distinguishing CD from ITB through interpretable machine learning, which can obtain a global explanation but also an explanation for individual patients.

## Introduction

Distinguishing Crohn’s disease (CD) from Intestinal tuberculosis (ITB) is of key importance for gastrointestinal diseases field^1^. Currently, tuberculosis has become one of the major public health threats all over the world. It remains a major reason of incidence and mortality in developing countries^2^. Along with tuberculosis patients increase, the morbidity of ITB also increased. However, CD and ITB have overlapping features in clinical symptoms, radiologic, endoscopic and histological characteristics, especially the existence of granulomatous^3^. What’s more, in case of misdiagnosis of ITB, human health risk will enhance due to the unnecessary use of anti-tuberculosis drugs. In contrast, using steroid or immunosuppressive therapy to cure CD may cause the spread of tuberculosis^4^. Therefore, it’s highly essential to explore an effective method for differentiating CD from ITB.

Existing diagnostic tests are difficult to differentiate CD from ITB due to their low sensitivities, such as mycobacterial culture, polymerase chain reaction, acid-fast bacilli^5,6^. Antituberculous therapy (ATT) for 8–12 weeks was recommended in the Asia–Pacific guide^7^. However, ATT treatment may produce a series of side-effects and result in serious complications^8^. Therefore, a large number of researches were devoted to discovering specific and diagnostic characteristics that may help distinguish between CD and ITB. A total of 36 cases were used to analyze the intestinal wall and mesentery features of CD and ITB, and provide a guide for diagnosis^9^. Epstein el at. suggested that in people who are at risk for ITB a CD diagnosis should be made after careful clinical interpretation, radiological, endoscopic and histological features^10^. The ratio of visceral fat area and subcutaneous fat area is measured on computed tomography and used as a classify characteristic^11^. Limsrivilai et al. used clinical, endoscopic and pathology features to differentiate these two diseases^12^. Israrahmed et al. These studies indicate that some clinical presentation, radiological, endoscopic and histological features can improve the diagnostic accuracy of CD and ITB.

Additionally, statistical models and scoring systems were explored to distinguish between these two diseases based on various features and modes. The statistical theory has provided a great variety of methods, those were used to determine sensitivity indices and improve the diagnostic accuracy of CD and ITB^3,13^; The logistic regression model (LOG) is the most popular. LOG can provide estimates of a continuous probability of CD or ITB in the patients using two extreme values for the probability of disease: 0 for negative and 1 for positive^14^. Assumptions for multivariate normality and equal covariance matrices are relaxed in LOG. What’s more, the LOG model has the significant superiority of easy interpretation for its results, providing a straightforward probability for individual patients. With these advantages, LOG has gradually been regarded as a scoring method to diagnose diseases. However, those methods still have several limitations: (1) they may be challenging to imitate the complex nonlinear interaction between variables, and (2) they have a high sensitivity to abnormal values (3) they are difficult to solve the problem of imbalance.

Recently, machine learning (ML) has achieved state-of-art performance in the fields of economy, management, medical science, etc.^15^. In contrast to statistical methods, ML algorithms can model complex non-linear relationships between predictors and disease outcomes, achieving superior out-of sample performance. Moreover, ML algorithms has recently attracted strong interest in gastroenterology and has achieved promising results^16^. However, it no longer provides a parameter estimate that correlates the predictors with the output variables, resulting in low transparency. Also, the highest accuracy of data sets is usually obtained through complex models that are difficult to explain for experts at the moment, such as ensemble learning or deep learning. In many applications, particularly in medical applications, understanding why a model makes a forecasting is as essential as the accuracy of the forecasting. Thus, the acceptance and application of ML models are still low and limited.

In return, several approaches have been developed to assist people in comprehending the results of complex model^17^. Among them, the tree-based global method of interpretation has a rich history, summarizing the impact features on the model as a whole^18^. Besides, due to the influence of model mismatch (low fitting capacity of linear models), a tree-based model is easier to explain than a linear model^19^. Nevertheless, simply reporting the path of a forecast is of little significance for most models that ignore abundant local information. In other words, it fails to pay attention to the impact of input characteristics on a single prediction (a single sample)^20^. Particularly in the medical domain, certain characteristics may not be of high global significance, but may be extremely important for specific individuals because of the heterogeneity of patients.

Based on the analysis, we propose an explainable machine learning framework for distinguishing CD from ITB through the clinical presentation, endoscopy and biochemical data. The proposed framework consists of three components. The first level performs the imbalanced treatment of the dataset using a SMOTE algorithm^21^. In the second level, a tree-based model is applied to detect CD from ITB. At the last level, the interpretation and visualization of the model are demonstrated through Shapley values^22^. To validate the superiority of the proposed approach, we compare the performance of six different classical algorithms, including Latent Dirichlet Allocation (LDA), Logistic Regression (LOG), Support Vector Machine (SVM), Artificial Neural Network (ANN), Radom Forest (RF) and Adaptive Boosting (Adaboost)^23–28^. The main contribution of this research is as follows: (1) This paper proposed an effective framework to addresses a real-world problem, differentiating CD from ITB; (2) This framework can improve the predictive performance combing with SMOTE algorithm and machine learning; (3) Our framework provide local interpretation and direct results of visualization without losing the classification accuracy based on a model-independent interpretable machine learning algorithm; (4) As for as we know, it is the first time to develop a interpretable machine learning framework to distinguish CD from ITB, which may improve medical workers’ acceptance of prediction outcomes.

## Materials and methods

### Data source and feature selection

The study was approved by the Ethics Committee of the 2nd Xiangya Hospital, Central South University. All experiments were performed in accordance with relevant guidelines and regulations. Informed consent was obtained from the subjects for the participation in the study. Intestinal data were collected from the Second Xiangya Hospital of Central South University, 160 patients with CD and 40 patients with ITB were included in the study. All the patients were with active disease. All cases were combined with the clinical diagnosis and European diagnostic guidelines of CD and ITB^29^. The CD diagnosis is according to clinical, endoscopic and pathological characteristics, as well as the clinical response to Crohn’s treatment. The diagnostic criteria of ITB include the following conditions: (1) there were acid-fast bacillus (AFB) or caseous granuloma in pathological diagnosis; (2) clinical recovery was complete with endoscopic mucosal healing and at least six months of antituberculosis therapy. After treatment, endoscopic follow-up was performed for 2–6 months. The institutional review committees of the participating centers have given their approval to this work.

We focus on the integration of basic parameters, including demographic data, clinical manifestations, biochemical indicators and endoscopic performance. The variables included are widely available in the diagnosis of Crohn and intestinal tuberculosis, which means that our diagnostic model has preferably general sense. The descriptive statistics of the dataset in paper are shown in Table 1.

**Table 1:** Demographic characteristics, clinical presentation, laboratory test, imaging characteristics between CD and ITB.

Category	CD	ITB	P value
**Demographic**			
Age, mean (SD)	31.59 ± 12.67	35.83 ± 13.94	0.071
Male (%)	117/160	31/40	0.479
**Clinical presentation**			
Intestinal surgery	63/160	9/40	< 0.05
Abdominal	138/159	13/31	< 0.01
Diarrhea	90/160	18/40	0.203
Bloody stool	37/157	2/39	< 0.05
Constipation	10/159	7/40	0.0668
OB	117/155	24/37	0.1946
Leukocyte	7.56 ± 2.70	6.80 ± 2.39	0.102
Neutral ratio	72.58 ± 10.38	68.81 ± 14.58	0.170
Hematocrit	36.16 ± 7.09	34.43 ± 7.35	0.312
Hemoglobin	112.34 ± 23.35	113.74 ± 24.38	0.587
Platelet	347.66 ± 121.75	333.95 ± 114.48	0.442
Tuberculosis history	7/160	14/40	0.593
**Biochemical index**			
PPD	4/97	11/22	< 0.01
IgM	2/92	0/26	0.506
IgG	28/88	9/26	0.631
Knot	4/90	5/26	< 0.05
ESAT-6	17/129	24/26	< 0.01
CFP-10	15/129	23/26	0.102
Chest radiology	52/131	7/34	0.472
ESR	35.82 ± 26.37	36.22 ± 20.71	0.086
CRP	40.94 ± 42.03	47.77 ± 33.27	0.086
**Imaging data**			
Albumin	33.02 ± 7.40	33.80 ± 6.85	0.484
Stratified reinforcement	136/146	29/39	0.140
Intestinal wall thickening	149/157	34/39	0.216
Intestinal stenosis	86/152	15/39	0.100
Intestinal dilatation	34/142	4/39	< 0.05
Inflammatory masses	2/156	4/39	0.077
Abscess	4/156	0/39	0.600
Lymphadenopathy	82/156	25/39	0.432
Comb sign	87/156	3/39	< 0.01

### SMOTE for imbalance data

Class imbalance is a challenging issue in data mining and machine learning^30^. This study includes 160 CD patients and 40 ITB patients, the imbalance rate reaches 1:4. Traditional models tend to predict the sample as the category with the majority of samples. Therefore, an unbalanced dataset learning method is considered in this paper.

Sampling technology, ensemble method and cost-sensitive learning are the most widely used approaches to resolving the issue of class imbalance^31^. Cost-sensitive learning allocates misclassification costs to different classes^32^. Generally, the cost of a few samples is high, while most samples are low. However, due to the accuracy of classification cost is difficult to obtain, results of the cost-sensitive learning method are usually unstable^33^. The methods based on sampling technology are still the mainstream of unbalanced data processing. The sampling approaches are utilized to alter the original class distribution through over-sampling the minority class or under-sampling the majority class instances. Nevertheless, resampling for majority classes may be a potentially useful training instance, while undersampling may not significantly improve the recognition for minority classes^34^. Rayhan proposed the Cusboost algorithm based on clustering sampling and compared it with several popular methods, including SMOTEboost and Rusboost. Each sampling method has its advantages Through the experiments on 19 public datasets^35^. Considering the small sample data used in this paper, we adopt the SMOTE algorithm for imbalanced data. The basic idea of SMOTE algorithm is to generate a few new samples by KNN technology and combine them with the original dataset^21^. This algorithm can be described as the following processes:

Consider a training dataset $D={\left\{{\mathbf{x}}_{i},{y}_{i}\right\}}_{1}^{m}$, ${\mathbf{x}}_{i}\in {\mathbb{R}}^{d}$ and ${y}_{i}\in {\mathbb{R}}^{l}$.

Step 1: Calculate each minority sample’s k nearest neighbors of using the KNN algorithm.
Step 2: To produce new sample points, N samples are randomly extracted from k nearest neighbors using random linear interpolation.
  1 $${x}_{\text{new }}={x}_{i}+\varepsilon \times \left({x}_{j}-{x}_{i}\right)$$
  where, ${x}_{i}$ denotes one of the minority samples, ${x}_{j}$ denotes its neighbor sample and ${x}_{new}$ express the new samples.
Step 3: Combine the new samples with original samples to generate a new training dataset.
  To achieve the balance of each epoch in the training process, the over-sampling rate is determined as follows:
  $$oversampling{\text{-}}rate=\frac{{N}_{major}}{{N}_{\mathit{min}or}}-1$$
  Here, ${N}_{major}$ and ${N}_{\mathit{min}or}$ denote the number of major class and minor class respectively.

### XGBoost algorithm

XGBoost is one machine learning algorithm that shines in practice, which has been yielded state-of-art performance in many industries^36^. In this chapter, we briefly describe the Xgboost algorithm.

Given a dataset with m features and n samples $D=\left\{{\mathbf{x}}_{i},{y}_{i}\right\}$, $\left|D\right|=n$, ${x}_{i}\in {\mathbb{R}}^{m}$, ${y}_{i}\in {\mathbb{R}}$. Regularization objective of XGBoost algorithm is:

2 $$Obj\left(\phi \right)=\sum_{i=1}^{n}l\left({\widehat{y}}_{i},{y}_{i}\right)+\sum_{k}\Omega \left({f}_{t}\right)$$

where $l$ is a differentiable convex loss function to measure the difference between the predicted value ${\widehat{y}}_{i}$ and the target value ${y}_{i}$. And the $\Omega \left(f\right)=\gamma T+\frac{1}{2}\lambda \parallel w{\parallel }^{2}$ denotes the penalty of model. In detail, $T$ is the number of leaves in the tree and the leaf weights are denoted by $w$. This term $\Omega$ penalizes the complexity of the model. The additional regularization term helps to smooth the final learnt weights to avoid over-fitting. In order to use traditional methods of optimization in Euclidean space, the model is trained in an additive way. Formally, the model greedily adds ${f}_{t}$ which can improve the most according to Eq. (2), then the objective can be expressed as follows.

3 $$Ob{j}^{\left(t\right)}=\sum_{i=1}^{n}l\left({y}_{i},{\widehat{y}}_{i}^{\left(t-1\right)}+{f}_{t}\left({{\varvec{x}}}_{i}\right)\right)+\Omega \left({f}_{t}\right)$$

where ${\widehat{y}}_{i}^{\left(t\right)}$ indicates the prediction of the $i$-th at $t$-th iteration. Utilizing Taylor’s second-order expansion, the objective of optimization can be written as follows.

4 $$Ob{j}^{\left(t\right)}\simeq \sum_{i=1}^{n}\left[l\left({y}_{i},{\widehat{y}}^{\left(t-1\right)}\right)+{g}_{i}{f}_{t}\left({{\varvec{x}}}_{i}\right)+\frac{1}{2}{h}_{i}{f}_{t}^{2}\left({{\varvec{x}}}_{i}\right)\right]+\Omega \left({f}_{t}\right)$$

Here ${g}_{i}={\partial }_{{\widehat{y}}^{\left(t-1\right)}}l\left({y}_{i},{\widehat{y}}^{\left(t-1\right)}\right)$ and ${h}_{i}={\partial }_{{\widehat{y}}^{\left(t-1\right)}}^{2}l\left({y}_{i},{\widehat{y}}^{\left(t-1\right)}\right)$ are the first and second-order gradient statistics of the loss function, respectively.

A simplified objective at iteration $t$ can be obtained through the traditional GBDT training process.

5 $${\tilde{Obj }}^{\left(t\right)}=\sum_{i=1}^{n}\left[{g}_{i}{f}_{t}\left({{\varvec{x}}}_{i}\right)+\frac{1}{2}{h}_{i}{f}_{t}^{2}\left({{\varvec{x}}}_{i}\right)\right]+\Omega \left({f}_{t}\right)$$

The optimal value for a fixed structure $q\left(\mathbf{x}\right)$ can be calculated by

6 $${\tilde{Obj }}^{\left(t\right)}\left(q\right)=-\frac{1}{2}\sum_{j=1}^{T}\frac{{\left(\sum i\in {I}_{j}{g}_{i}\right)}^{2}}{\sum i\in {I}_{j}h+\lambda }+\gamma T$$

By expanding Taylor’s second-order loss term of objective function, XGBoost retains more information. Simultaneously, the regularization term of branch node weight was utilized to improve the model’s performance. In this paper, the XGBoost algorithm is used to train a model for distinguishing CD and ITB.

### Explainability for machine learning

Understanding why a mathematical model makes a certain prediction can is of significance in many applications, especially in medical science^37^. A key factor in whether doctors will use machine learning model prediction for clinical decision-making is how they can know how the model makes a prediction. The definition of interpretability is to help people understand the reason and degree of machine learning prediction. Although the machine learning algorithm can model the complex nonlinear between variables, it can no longer provide the parameter estimation associated with predictors and result variables and has low transparency.

In this paper, we introduce the Shapley additional explanations (Shap) method to explain our machine learning model^38^. Shap approach is an additive interpretative model inspired by cooperative game theory. All the features are regarded as contributors, and it has been proved consistent with the importance of features in theory.

To better describe the Shap approach, we first introduce a significant concept call Shapley value, which can allocate the cooperation benefit fairly by considering the contribution of each agent. Shapley value of agent $i$ is equal to the average value of the expected contribution of which for a cooperation project. Suppose a cooperation program $C=\langle Ag,v\rangle$, including several agents ($Ag=\{1,2,\ldots ,n\},n\ge 2$) and a characteristic equation $v(C)=k(\ge {\sum }_{i\in C}{x}_{i})$ of each agent’s contribution to this project^39^.

Define the marginal contribution of agent $i$ joining the organization $S$ as:

7 $${\delta }_{i}(S)=v(S\cup \{i\})-v(S)$$

Then the Shapley value of agent $i$ can be expressed as follow:

8 $$Sh(S,i)/{\varphi }_{i}={\sum }_{r\in R}{\delta }_{i}({S}_{i}(r))/\left|Ag\right|!$$

Corresponding to the interpretation of machine learning prediction, ‘game’ refers to the prediction task of a single instance, ‘revenue’ denotes the predicted value of the instance minus the average predicted value of all instances, while ‘player’ refers to the instance’s features, and they work together to obtain income.

Consider the contribution of each feature to the outcome. It is straightforward to obtain the effect in the linear model. The prediction of a data instance’s linear model can be depicted as:

9 $$\stackrel{\wedge }{f}(x)={\beta }_{0}+{\beta }_{1}{x}_{1}+\cdots +{\beta }_{p}{x}_{p}$$

where $x$ denotes the instance. ${x}_{j},j=1,\ldots ,p$ states the feature of each instance. ${\beta }_{j}$ is the weight corresponding to ${x}_{j}$. The contribution of $j$-th feature to prediction $\stackrel{\wedge }{f}(x)$ is reported as ${\phi }_{j}$.

10 $${\phi }_{j}(\stackrel{\wedge }{f})={\beta }_{j}{x}_{j}-E({\beta }_{j}{X}_{j})={\beta }_{j}{x}_{j}-{\beta }_{j}E({X}_{j})$$

where $E({\beta }_{j}{X}_{j})$ denotes the average estimated effect value, that is, the contribution is the difference between characteristic effect and average effect.

Each feature’s Shapley value is the weighted amount of its total expenditure (prediction) over all possible combinations of features.

11 $${\phi }_{j}(v)={\sum }_{S\subseteq \{{x}_{1},\ldots ,{x}_{p}\}\backslash \{{x}_{j}\}}\frac{\left|S\right|!(p-\left|S\right|-1)!}{p!}(v(S\cup \{{x}_{j}\})-v(S))$$

where $S$ is a subset of the features used in the model. $x$ denotes the vector of the instance’s feature to be interpreted, while the number of features is recorded as $p$. ${v}_{x}(S)$ expresses the prediction of features in set $S$, which is the marginalization of features not included in the set $S$.

12 $${v}_{x}(S)=\int \stackrel{\wedge }{f}({x}_{1},\ldots ,{x}_{p})d{\text {P}}_{x\notin S}-{E}_{X}(\stackrel{\wedge }{f}(X))$$

Actually, Eq. (12) performs multiple integrals for each feature that is not include. It is worth noting that the Shapley value of the feature $j$ is explained as follows: compared with the average prediction of the dataset, the contribution of the $j$-th feature to the prediction of this feature instance is ${\varphi }_{j}$. Therefore, the Shapley value of the feature is not the difference of the predicted value after deleting the feature from the model, which can be regarded as the definition of fair expenditure.

Shap method can effectively estimate Shapley value according to the local agency model. This method can quickly implement a tree-based model through linking LIME and Shapley^20^. Shap defines the interpretation as:

13 $$g({z}^{^{\prime}})={\phi }_{0}+\sum \limits_{j=1}^{M}{\phi }_{j}{z}_{j}^{^{\prime}}$$

where $g$ is the interpretation model, ${z}^{^{\prime}}\in \{\mathrm{0,1}{\}}^{M}$ is the alliance vector and $M$ denotes the size of the largest alliance. The Shapley value of feature $j$ is recorded as ${\phi }_{j}\in {\mathbb{R}}$. In the alliance vectors, 1 means the corresponding feature exists, while 0 expresses it does not exist. As for interested instance $x$, all of the alliance vectors are equal to 1, which means that features exist. A can be simplified as:

14 $$g({x}^{^{\prime}})={\phi }_{0}+\sum \limits_{j=1}^{M}{\phi }_{j}$$

In theory, Shapley value is the only solution that satisfies efficiency, symmetry, virtuality and additivity. Shap method also meets the conditions, which can calculate shapely values. Specifically, the shap method describes the properties of the following three ideals:

**Missingness.**

15 $${x}_{j}^{^{\prime}}=0\Rightarrow {\phi }_{j}=0$$

Missingness means that the attribute of missing feature is zero. ${x}_{j}^{^{\prime}}$ denote the alliance, a value of zero indicates that a feature in this instance is missing. Theoretically, a missing feature can have any Shapley value without compromising local accuracy because it is multiplied by ${x}_{j}^{^{\prime}}=0$. This property forces the missing feature to obtain a Shapley value of zero.

**Additivity.**

16 $$f\left(x\right)=g\left({x}^{^{\prime}}\right)={\phi }_{0}+\sum_{j=1}^{M}{\phi }_{j}{x}_{j}^{^{\prime}}$$

Additivity is also called local accuracy. It implies that the outcome of the model to be explained is equal to the sum of feature’s attribute. Where ${\phi }_{0}={E}_{X}\left(\widehat{f}\left(x\right)\right)$ , that is, the average of predicted values of the model.

**Consistency.**

Let ${f}_{x}\left({z}^{\mathrm{^{\prime}}}\right)=f\left({h}_{x}\left({z}^{\mathrm{^{\prime}}}\right)\right)$ and ${z}_{\setminus {j}^{\mathrm{^{\prime}}}}$ denotes that ${z}_{j}^{\mathrm{^{\prime}}}=0$. For any two models $f$ and ${f}^{\mathrm{^{\prime}}}$, any ${z}^{^{\prime}}\in \{\mathrm{0,1}{\}}^{M}$:

17 $${f}_{x}^{^{\prime}}\left(z\right)-{f}_{x}^{^{\prime}}\left(z\setminus j\right)\ge {f}_{x}\left(z\right)-{f}_{x}\left(z\setminus j\right)$$

satisfy $${\phi }_{j}\left({f}^{\mathrm{^{\prime}}},x\right)\ge {\phi }_{j}\left(f,x\right)$$.

Consistency means that if the marginal contribution of the feature increases or remains unchanged due to the change of the model, the Shapley value will increase or remain unchanged accordingly. In Eq. (17), function ${h}_{x}({z}^{^{\prime}})=z$, ${h}_{x}:\{\mathrm{0,1}{\}}^{M}\to {\mathbb{R}}^{p}$, which is used to convert the alliance of features into effective data instances. That is, the corresponding value mapped to the instance $x$ we want to interpret.

Therefore, the global contribution of the variable can be calculated using the Shap method's local contribution. We average the Shapley absolute value of each feature in the dataset.

18 $${I}_{j}=\sum \limits_{i=1}^{n}\left|{\phi }_{j}^{(i)}\right|$$

## Simulation experiment

We initially obtained clinical data with 32 features from 200 patients (CD = 160, ITB = 40). This dataset is used to train the proposed method as well as the comparative approaches.

### Study design

**Figure 1:**
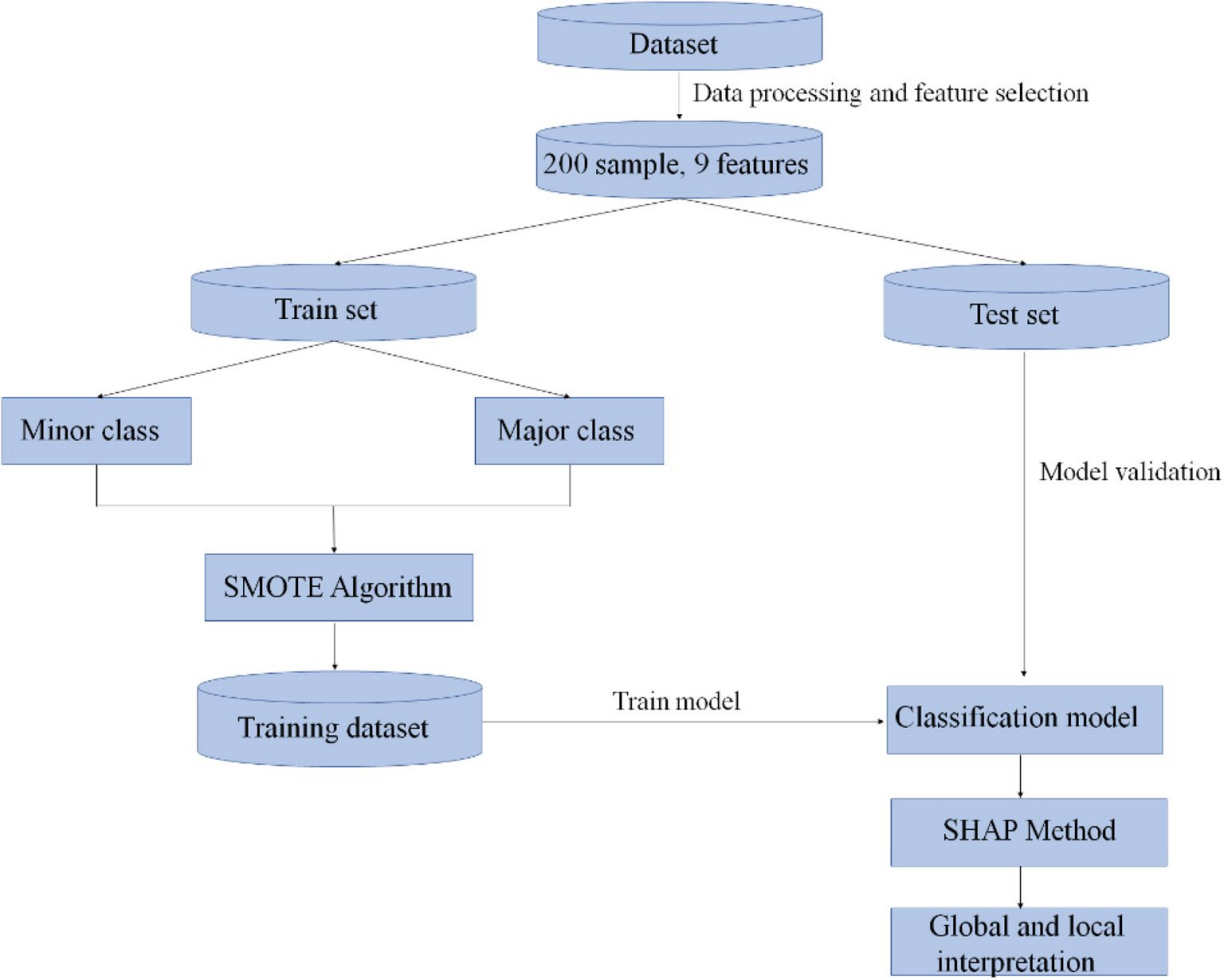
Overview of the proposed framework.

Figure 1 demonstrates the overview of proposed framework using an explainable machine learning method. It mainly includes data preprocessing, model input feature selection, imbalance category processing, model establishment and interpretation.

Before building the machine model, the significance test method is applied to obtain significant features related to the target. Compared with t-test, Mann–Whitney U test is appropriate for small samples, and does not require data correspond to normal distribution. Therefore, Mann–Whitney U test is used to select continuous variables related to CD and ITB identification. Chi-square test was used for categorical variables. Then, the average method is utilized to deal with missing values of continuous variables, while the counting variables with missing values are marked with other numbers.

Based on this, a total of nine features are chosen as input variables of the classification model. In details, the cohort consisting of 200 samples was stratified random sampling, splitting into 2 datasets—training set (60%) and testing set (40%). Next, upsampling the minor class instances in the training set through SMOTE algorithm. Among which, the training set was used to train a classification model for distinguishing CD from ITB, and the evaluation metrics of methods were reported on thetoprule testing set. Finally, the SHAP method was introduced to explain the output of the model.

### Evaluation criteria

Differentiating CD from ITB is a binary classification problem. We choose five different functions to evaluate the performance of models, including sensitivity, specificity, precision, area under the receiver operating characteristic curves (AUC) and Matthews correlation coefficient (MCC). Suppose that the instance ITB is a positive class and the instance CD is a negative class. According to the confusion matrix, these criteria can be described as follows:

19 $$\text{Sensitivity }=\frac{TP}{TP+FN}$$

20 $$\text{Specificity }=\frac{TP}{TN+FP}$$

21 $$\text{Precision }=\frac{TP}{TP+FP}$$

The value of AUC is between 0 and 1, which can directly evaluate the quality of the classifier. The larger AUC value denotes the better performance of a classifier.

Besides, MCC is introduced in this paper, which is a balanced evaluation criterion applicable to the unbalanced category.

22 $$MCC=\frac{TP\times TN-TP\times FN}{\sqrt{\left(TP+FP\right)\times \left(TP+FN\right)\times \left(TN+FP\right)\times \left(TN+FN\right)}}$$

MCC essentially describes the correlation coefficient between the predicted results and the actual values.

### Implementation details

Significant indicators were included in classification models through the Mann–Whitney U test and Chi-square test, including Intestinal surgery, Abdominal, Bloody stool, PPD, Knot, ESAT-6, CFP-10, Intestinal dilatation and Comb sigh (see Table 1).

In this paper, the XGBoost algorithm is compared with two statistical methods and several machine learning. For instance, linear discriminant analysis (LDA), logistic regression (LOG), artificial neural network (ANN), support vector machine with different kernel functions, Bayesian regression (Bayes), random forest (RF) and gradient boosting decision tree (GBDT). Among which, as statistical methods, LDA and LOG are usually employed to solve a binary classification problem. ANN, SVM and Bayes are classic machine learning models based on different theories, which are commonly utilized as benchmark methods of machine learning. Besides, RF and GBDT are considered, two significant approaches in the development of the tree model.

The unbalanced rate of CD and ITB is used to over-sample the minor class instances (ITB), to achieve the balance of each epoch in the training process.

As for machine learning models, whose hyperparameters define the general characteristics, may directly affect its prediction accuracy. Therefore, it’s extremely significant to optimize them. In particular, considering the complexity of ANN, this paper uses the single hidden layer network structure. The number of hidden layer neurons is important for the ANN model. Similarly, the optimal penalty coefficient C of SVM-linear, SVM-sigmoid and SVM-RBF is also obtained by cross-validation. For the three tree models (RF, GBDT and XGBoost), the most important parameters are the number of trees and the max feature. A larger number of trees would improve the performance of models, with more calculation cost. What’s more, the prediction accuracy would no longer improve if the number of trees exceeds the special value. The max feature is determined by the features of the square root. The number of trees is optimized by cross-validation and the other parameters are gained by default values. In detail, parameter k for k-nearest neighbors of SMOTE algorithm is choose as 3. The regularization parameter $\lambda$, learning rate, number of trees and the max feature are ultimately determined as 0.01, 0.1, 100 and 5. Other hyperparameters are the default values. All of parameters are ultimately determined by fivefold cross-validation. To make the result more reliable, each model is run 500 times to obtain an integrated average forecast. All analyses were carried out using Python, version 3.6.5 on a Dell server with 16 GB RAM.

### Results and analysis

The comparison between different classifiers illustrates that the XGBoost algorithm yields a promising performance with a mean AUC of 0.891. It also outperforms other classification models in terms of sensitivity, specificity, precision and MCC (see Table 2). Naive denotes the model without applying the SMOTE algorithm, which means does not use the class imbalance method. Results depict that applying SMOTE algorithm can improve the prediction performance.

**Table 2:** Performance of different methods for distinguishing CD from ITB.

Model	AUC		Sensitivity		Specificity		Precision		MCC	
	Naive	Our	Naive	Our	Naive	Our	Naive	Our	Naive	Our
LDA	0.750	0.785	0.563	0.623	0.938	0.948	0.692	0.761	0.542	0.617
LOG	0.766	0.806	0.625	0.735	0.906	0.877	0.625	0.631	0.531	0.583
ANN	0.736	0.778	0.605	0.641	0.921	0.915	0.676	0.672	0.549	0.567
SVM-linear	0.773	0.798	0.688	0.754	0.859	0.842	0.550	0.560	0.505	0.641
SVM-sigmoid	0.625	0.670	0.375	0.701	0.875	0.638	0.429	0.330	0.263	0.227
SVM-rbf	0.812	0.841	0.750	0.787	0.875	0.895	0.600	0.662	0.577	0.641
Bayes	0.809	0.820	0.753	0.750	0.866	0.891	0.649	0.632	0.598	0.602
RF	0.829	0.844	0.702	0.734	0.956	0.955	0.625	0.817	0.699	0.717
GBDT	0.839	0.849	0.726	0.749	0.951	0.969	0.803	0.801	0.704	0.716
XGBoost	0.853	0.891	0.752	0.813	0.953	0.969	0.818	0.867	0.729	0.801

Among these methods, RF and GBDT are second only to the XGBoost algorithm. Notably, the performance of specificity is superior to sensitivity in most models. In this work, specificity indicates the probability to detect CD correctly while sensitivity expresses the extent to correctly detect ITB. This may mean that the identification of ITB is more challenging. To sum up, compared with traditional statistical methods, the machine learning algorithm performs better in the classification of CD and ITB.

**Figure 2:**
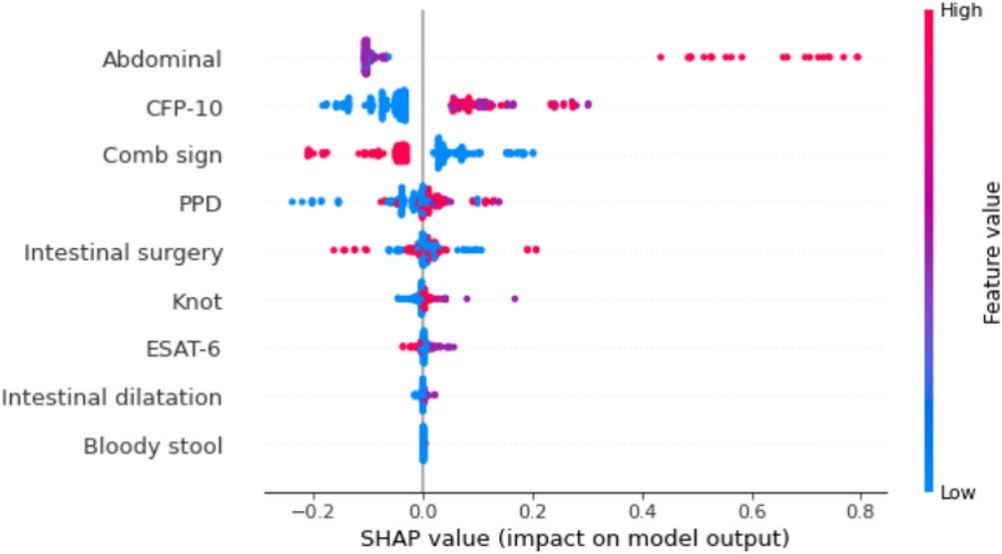
SHAP summary plot for the XGBoost algorithm.

Figure 2 shows the SHAP summary plot for the XGBoost model. In our experiment, CD and ITB are coded as 0 and 1 respectively. Each point in the figure represents a sample, that is, the patient. The horizontal coordinate represents the Shapley corresponding to each feature of each sample. A positive value indicates that the prediction probability of ITB would be improved. The corresponding negative value denotes that the prediction probability is reduced, which means the probability of CD would be increased. The color in the plot represents the value of the feature, red denotes the feature with a large value, while blue represents a feature with a small value. For a binary variable, red color denotes 1 (positive) and blue color denotes 0 (negative). For instance, the Shapley value of the majority red sample in CFP-10 feature, indicating that positive CFP-10 would improve the probability of ITB patients. The majority blue sample of CFP-10 denotes that negative would improve the probability of CD patients. In term of color discrimination, Abdominal, CFP-10 and comb sign can be more effective to distinguish CD and ITB. Besides, the long right tails of abdominal in the summary plot mean that it is rare but may a high-magnitude risk factor.

It should be emphasized that a feature with low differentiation does not mean that it is not important, which reflects some characteristics that may not occur in these two diseases. The main advantage of the SHAP summary plot is that the effect of different variables on the prediction can be in a highly visual way.

**Figure 3:**
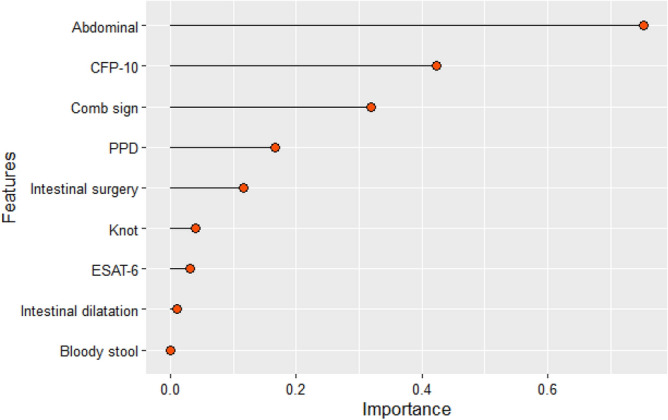
Bar chart plot for the nine significant variables contributing to the XGBoost model’s prediction for distinguishing CD from ITB.

We can use the SHAP method to gain a global explanation for our prediction (calculated by Eq. 22). The global explanations are obtained by calculating the SHAP explanations for all individual patients and then averaging them per feature. Figure 3 gives the bar chart plot for the nine significant variables contributing to the XGBoost model’s forecasting for identifying CD and ITB. The greater the length, the greater the importance of the variable. It can be seen that features abdominal, CFP-10, Comb sign, PPD and intestinal surgery are more important predictors to distinguish CD from ITB.

**Figure 4:**
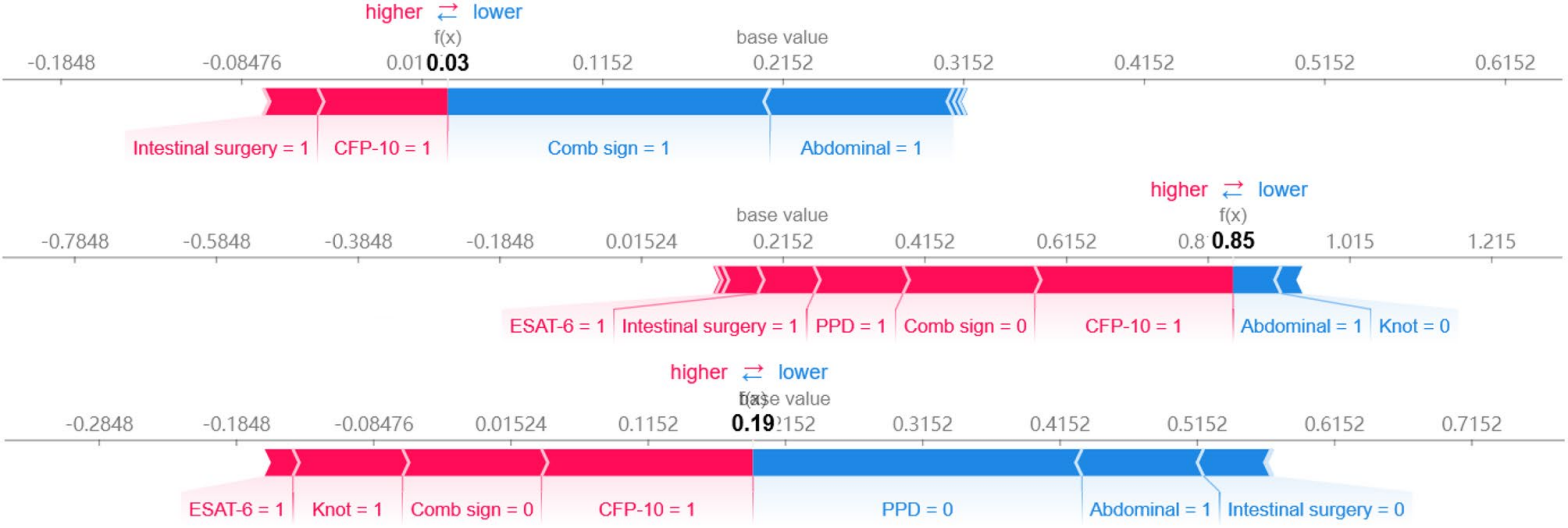
SHAP explanation plot for three patients from our testing dataset.

More importantly, the SHAP method can visualize the effect of input variables on each patient (see Fig. 4). The base value is the average of all predicted values of the model on the testing set. Features with red color and blue color indicate that increases (positive values) and decreases (negative value) the prediction compare with its baseline.

The first patient was diagnosed with CD, and the probability of CD predicted by the model is 0.97 (1–0.03). Intestinal surgery, CFP-10, comb sign and abdominal of this patient are positive. Among which, comb sign and abdominal decrease the prediction of ITB while the others increase. For him, the comb sign and abdominal play a more important role in decreasing the prediction. That’s why the model diagnosed him as a CD.

The second patient is an ITB with a prediction probability of 0.85. As for this patient, abdominal (positive) and knot (negative) decrease the prediction of ITB. However, ESAT-6 (positive), intestinal surgery (positive), PPD (positive), comb sign (negative) and CFP-10 (positive) play a more important role in increasing the prediction probability.

The third patient was diagnosed with CD with a prediction probability of 0.81 (1–0.19). ESAT-6, Knot, CFP-10, and Abdominal of this patient are positive, while the Comb sign, PPD, intestinal surgery are negative. In details, PDD, Abdominal and Intestinal surgery increase the prediction of CD while the others decrease. It is worth noting that the effects of the same characteristics on different individuals are different. For example, CFP-10 (positive) in the first patient and third patient increase the prediction of ITB, while the contribution intensity is different (corresponding to the length of the color in the figure).

## Discussion

In recent years, the morbidity of CD has increased significantly with the industrialization of many countries. Meanwhile, the incidence of intestinal tuberculosis (ITB) has been increasing. Distinguishing Crohn’s disease (CD) from intestinal tuberculosis (ITB) has always been a challenge for clinicians in developing countries. PPD and the tuberculosis (TB) interferon-gamma (IFN-γ) release assay (TB-IGRA) are both associated with mycobacteria, and have a relatively high sensitivity and specificity for the diagnosis of ITB, especially TB-IGRA. However, in addition to active TB infection, individuals with latent infection or past infection can also have a positive result of TB-IGRA and PPD. As we know that tuberculosis is still prevalent in developing countries, so positive result of TB-IGRA and PPD can be detected in a considerable proportion of the population in China, including some of the CD patients. Since IGRA and PPD have some difficulties in distinguishing active TB infection from latent or past TB infection, empirical anti-tubercular therapy (ATT) trial, and subsequent clinical and endoscopic response to ATT is still required in a significant proportion of patients to confirm the diagnosis. In our research, some of the CD patients with insufficient phenotype and positive PPD or TB-IGRA were established the final diagnosis of CD by empirical anti-TB therapy. However, ATT trial is associated with a delay in the diagnosis of CD, which may lead to poor prognosis and even serious side effects. Better method for improved differentiation is needed to reduce the need for ATT trial. Many researchers have been established several models to address this issue by using the clinical symptoms, laboratory tests, endoscopic findings and so on^3,9–12,40^. Israrahmed et al. conduct a prospective study, which proposed multiple variables to arrive at the final diagnosis of CD and ITB^41^. Especially, Kim et al. develop a deep-learning system for differentiation between Crohn’s disease, intestinal Behcet’s disease (BD) and intestinal tuberculosis using 6617 colonoscopy images of 211 CD, 299 BD and 217 ITB patients^42^. It is undeniable that the larger the sample size, the more meaningful the results are. However, those studies didn’t pay enough attention to the interpret of diagnostic models, especially the machine learning.

In terms of methods, the logistic regression model is one of the most popular in the field of healthcare. It’s easy to understand the results through weights in the equation. Nevertheless, the logistic model is usually not good from the perspective of prediction ability. The comparison between different classifiers illustrates that the XGBoost algorithm yields a promising performance with a mean AUC of 0.891. It also outperforms other classification models in terms of sensitivity, specificity, precision and MCC. Among these methods, RF and GBDT are second only to the XGBoost algorithm. Notably, the performance of specificity is superior to sensitivity in most models. In this work, specificity indicates the probability to detect CD correctly while sensitivity expresses the extent to correctly detect ITB. This may mean that the identification of ITB is more challenging. Also, the explanation of weight is not intuitive when the correlation or complex relationship occurs in variables. Prior studies have noted the advantages of machine learning methods in fitting the complex relationship between predictors and targets. Thus, machine learning can perform better than traditional statical methods in out-of samples. However, their prediction results are difficult to be accepted by medical faculty due to the low transparency, although many researchers are devoted to helping people understand how machine learning models make such predictions. For instance, partial dependence plots, accumulated local effects, feature interaction, feature importance, global surrogate models and tree models. There are still several limitations among these methods: (1) only single feature can be explained effective; (2) independence condition must be satisfied; (3) only the global interpretation can be obtained. Besides, all of them have no solid theory that the contribution of features can’t be calculated reasonably. In our study, the model’s interpretability makes it possible to determine the contribution rate of a single variable in the prediction, which reflects the individualization of the prediction model. To sum up, compared with traditional statistical methods, the machine learning algorithm performs better in the discrimination of CD and ITB.

SHAP, a method to explain an individual prediction, is the only explanation approach with solid theory at present. This method was also used to predict GI bleed mortality in the intensive care unit, which yield a proposing performance^43^. Base on this, we propose an interpretable machine learning framework for distinguishing CD from ITB, which combing SMOTE algorithm and XGBoost method with SHAP method. Results prove that machine learning is superior to the traditional methods for differentiating CD and ITB. What’s more, the SHAP method can effectively obtain a global explanation but also an explanation for individual patients. This work may improve medical workers’ acceptance of prediction outcomes by machine learning without sacrificing accuracy.

Nonetheless, there are certain limitations to this paper. Because all the samples in our research were collected from a single center, the sample size, especially the ITB patients are somewhat small due to the limitation of clinical reality. Further research is required to establish the framework through more samples and include more variables.
